# Generation and Functional Characterization of Monocytes and Macrophages Derived from Human Induced Pluripotent Stem Cells

**DOI:** 10.1002/cpsc.108

**Published:** 2020-03-11

**Authors:** Xu Cao, Francijna E. van den Hil, Christine L. Mummery, Valeria V. Orlova

**Affiliations:** ^1^ Department of Anatomy and Embryology Leiden University Medical Center Leiden The Netherlands; ^2^ Department of Applied Stem Cell Technologies University of Twente Enschede The Netherlands

**Keywords:** differentiation, functional characterization, induced pluripotent stem cells, macrophages, monocytes

## Abstract

Monocytes and macrophages are essential for immune defense and tissue hemostasis. They are also the underlying trigger of many diseases. The availability of robust and short protocols to induce monocytes and macrophages from human induced pluripotent stem cells (hiPSCs) will benefit many applications of immune cells in biomedical research. Here, we describe a protocol to derive and functionally characterize these cells. Large numbers of hiPSC‐derived monocytes (hiPSC‐mono) could be generated in just 15 days. These monocytes were fully functional after cryopreservation and could be polarized to M1 and M2 macrophage subtypes. hiPSC‐derived macrophages (iPSDMs) showed high phagocytotic uptake of bacteria, apoptotic cells, and tumor cells. The protocol was effective across multiple hiPSC lines. In summary, we developed a robust protocol to generate hiPSC‐mono and iPSDMs which showed phenotypic features of macrophages and functional maturity in different bioassays. © 2020 The Authors.

**Basic Protocol 1**: Differentiation of hiPSCs toward monocytes

**Support Protocol 1**: Isolation and cryopreservation of monocytes

**Support Protocol 2**: Characterization of monocytes

**Basic Protocol 2**: Differentiation of different subtypes of macrophages

**Support Protocol 3**: Characterization of hiPSC‐derived macrophages (iPSDMs)

**Support Protocol 4**: Functional characterization of different subtypes of macrophages

## INTRODUCTION

Monocytes and macrophages play crucial roles in protective immunity and tissue hemostasis and trigger or exacerbate many pathological conditions, including diabetes, atherosclerosis, fibrosis, and cancer (Wynn, Chawla, & Pollard, 2013). Human peripheral blood mononuclear cells (PBMCs) are widely used as a source of human monocytes and macrophages for biomedical studies. However, their availability is often limited especially from patients with rare genetic diseases and there may be significant donor‐to‐donor variability. In addition, PBMC‐derived macrophages (PBDMs) have a different developmental origin than tissue resident macrophages in many organs, which presents a shortcoming for their application in disease modeling in vitro (Ginhoux & Jung, 2014).

Previous studies have shown that human induced pluripotent stem cells (hiPSCs) can be induced to form hemogenic endothelium (HE), identified as CD144+CD34+ and CD73‐, which could be further differentiated into CD43+ hematopoietic progenitors (HPCs) and then to erythro‐myeloid progenitors (EMPs); these are reminiscent of EMPs in the yolk sac during embryonic hematopoiesis. The differentiation process is MYB‐independent and associated with HOXA expression (Buchrieser, James, & Moore, 2017; Dou et al., 2016; Ivanovs et al., 2017; Ng et al., 2016; Vanhee et al., 2015), indicating this recapitulates primitive hematopoiesis in vivo. Other studies found that hiPSC‐derived macrophages (iPSDMs) could acquire tissue‐specific characteristics in vitro (Takata et al., 2017) and in vivo (Happle et al., 2018; Takata et al., 2017) by coculture with other tissue‐resident cell types. All of these studies indicate that iPSDMs can be a unique source of patient‐specific, tissue‐resident macrophages given that they can be produced in unlimited cell numbers from a renewable donor source of choice and can adopt a tissue resident macrophage‐like identity.

This article describes two basic protocols: The efficient monolayer differentiation of monocytes from hiPSCs (Basic Protocol 1), and then induction of their differentiation to different macrophage subtypes from cryopreserved monocytes (Basic Protocol 2); this is an adaption of our previous publication (Cao et al., 2019). Using our protocol, functional monocytes and polarized macrophage subtypes can be efficiently derived from hiPSCs within 2 and 3 weeks, respectively.

All protocols described in this article have been tested using at least three hiPSC lines and demonstrated as highly reproducible (Cao et al., 2019). Prior to the initiation of the differentiation process, hiPSCs are cultured in chemically defined E8™ medium on human recombinant vitronectin‐coated plates and passaged routinely every week when ∼90% confluent. For the passaging, hiPSCs are dissociated with gentle cell dissociation reagent (GCDR) at room temperature to obtain small cell clumps. Cells are passaged with a split ratio of 1:10 to 1:20 for maintenance. Cells are refreshed 48 hr after seeding and then every 24 hr. For the differentiation, hiPSCs are passaged similarly as for maintenance on Matrigel‐coated plates in E8™ medium. Details of the differentiation process are described in Basic Protocols 1 and 2. Isolation and cryopreservation of hiPSC‐derived monocytes (hiPSC‐mono) are described in Support Protocol 1. Support Protocol 2 describes methods for characterization of hiPSC‐mono. Support Protocol 3 describes methods for characterization of iPSDMs. In Support Protocol 4, four different functional assays are described for the analysis of endocytosis, bacterial phagocytosis, efferocytosis, and tumor phagocytosis activities of iPSDMs.

## Basic Protocol 1 DIFFERENTIATION OF hiPSCs TOWARD MONOCYTES

This protocol describes methods for the efficient derivation of hiPSC‐mono in 14 to 15 days, which had been tested and proven robust with multiple hiPSC lines previously (Cao et al., 2019).

**Figure 1:**
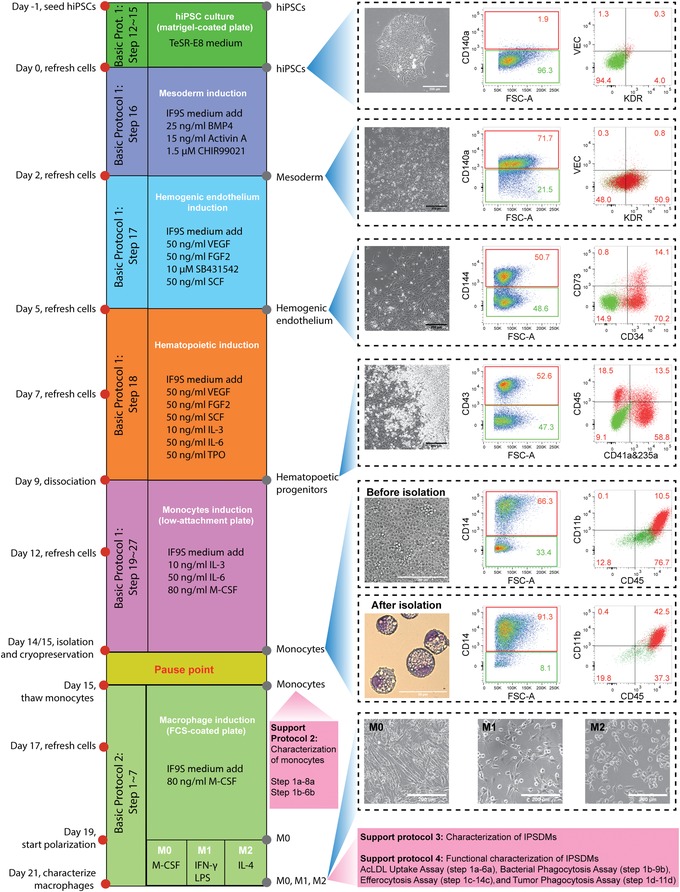
Timeline of all protocol procedures. Bright‐field images of representative cellular morphology are shown for day 0 (undifferentiated hiPSCs), day 2 (mesoderm), day 5 (HE), day 9 (HPCs), day 14 or 15 (monocytes before isolation), and day 21 (M0, M1, and M2 macrophages). FACS analysis of stage‐specific markers at day 0, day 2, day 5, day 9, and day 15 of differentiation are shown. Wright‐Giemsa staining of hiPSC‐mono after isolation at day 15 is also shown. Scale bar represents 200 μm in all bright‐filed images and 50 μm in Wright‐Giemsa staining. Abbreviations: hiPSCs, human induced pluripotent stem cells; HE, hemogenic endothelium; HPCs, hematopoietic progenitor cells; FACS, fluorescence activated cell sorting; hiPSC‐mono, hiPSC‐derived monocytes

Monocyte differentiation from hiPSCs is divided into five steps (Fig. 1). The first is seeding and culture of hiPSCs. The seeding density determines the differentiation efficiency so needs to be optimal because higher seeding densities can suppress HPC induction from day 5 to day 9. The second step is mesoderm induction with bone morphogenetic protein 4 (BMP4), activin A, and CHIR99021 for 2 days, followed by HE induction for 3 days with vascular endothelial growth factor (VEGF), basic fibroblast growth factor (FGF2), SB431542, and stem cell factor (SCF). On day 2 and day 5, the differentiation efficiency can be determined by fluorescence activated cell sorting (FACS) for mesoderm (CD140a+) and HEs (CD144+CD34+CD73‐), respectively. The fourth step is hematopoietic induction from day 5 to day 9 using VEGF, FGF2, SCF, interleukin 3 (IL‐3), interleukin 6 (IL‐6), and thrombopoietin (TPO). The differentiation efficiency can easily be estimated based on the number of round HPCs that emerge or are quantified by FACS for the HPC‐specific marker CD43 on day 9. The last step is monocyte induction from HPCs using IL‐3, IL‐6, and macrophage colony‐stimulating factor (M‐CSF) in suspension culture. On day 9, the round HPCs are collected first before dissociation of adherent cells using TrypLE and Accutase sequentially to minimize cell stress. Large numbers of dead cells and cell debris may lead to unintended monocyte activation. CD14+ monocytes can be harvested and isolated either on day 14 or day 15 depending on differentiation efficiency and/or on the hiPSC line, and optimal time can be determined either by FACS or by observing a small number of cells that start to adhere to the plate and differentiate towards macrophages.

Expected results are large numbers of round HPCs forming by day 9 of differentiation and >50% CD14+ monocytes before isolation. More details on expected results are described in the Understanding Results section.

Part or all of the protocol can be tailored to specific end‐user interests. For instance, the differentiation protocol for CD73‐ HEs from hiPSCs can be useful to study HE development in vitro and the subsequent endothelial‐to‐hematopoietic transition (EHT). HPCs derived from this protocol on day 9 show multilineage differentiation potential in a colony forming unit (CFU) assay, developing to erythroid, myeloid (granulocytes, monocytes, macrophages), and megakaryocyte lineages (Cao et al., 2019). Uenishi and colleagues showed that T lymphoid cells can also be generated from hiPSC‐derived HPCs differentiated using a comparable method, even though Tenascin C was used as an extracellular matrix protein to promote lymphoid differentiation (Uenishi et al., 2014). These results suggest that HPCs derived using this protocol can also be used for the induction of other hematopoietic lineages, including granulocytes, erythrocytes, megakaryocytes, and T lymphocytes.

### Materials

Human induced pluripotent stem cells (hiPSCs)
  LUMC0020 (LU20, generated from skin fibroblasts; Zhang et al., 2014);
  LUMC0054 (LU54, generated from kidney epithelial cells isolated from cells in urine, http://hpscreg.eu/cell-line/LUMCi001-A; Halaidych et al., 2018a); and
  LUMC0083 (LU83, generated from skin fibroblasts; Cao et al., 2019)
CellAdhere™ Dilution Buffer (Stemcell Technologies, cat. no. 07183)
Gentle Cell Dissociation Reagent (GCDR; Stemcell Technologies, cat. no. 07174)
TeSR™‐E8™ Kit for hESC/hiPSC (Stemcell Technologies, cat. no. 05990)
Vitronectin‐coated 6‐well plate (see recipe)
Matrigel‐coated 6‐well plate (see recipe)
IF9S medium (see recipe)
Mesoderm induction medium (see recipe)
Hemogenic endothelium induction medium (see recipe)
Hematopoietic induction medium (see recipe)
Monocyte induction medium (see recipe)
Accutase‐Solution (PromoCell, cat. no. C‐41310)
TrypLE™ Express Enzyme (1×), no phenol red (Gibco brand; Thermo Fisher Scientific, cat. no. 12604021)
Fluorescence activated cell sorting buffer (FACSB)‐10 (see recipe)
Dulbecco's phosphate‐buffered saline (DPBS)

Tubes, 15 ml (Greiner Bio‐One, cat. no. 188271)
Cell scraper, blue, 28 cm (Greiner Bio‐One, cat. no. 541070)
Costar® 24‐well Clear Flat Bottom Ultra‐Low Attachment Multiple Well Plates (Corning, cat. no. 3473)

### Passage hiPSCs for maintenance

1 Prewarm a vitronectin‐coated 6‐well plate, CellAdhere™ dilution buffer, and TeSR‐E8 at room temperature for at least 30 min.
2 Remove differentiated parts of hiPSC colonies by scraping them with a 200‐µl pipet tip.
3 Aspirate TeSR‐E8 containing differentiated parts of the culture.
4 Add 1 ml GCDR to each well of a 6‐well plate and incubate 5 min at room temperature.
  The timing of GCDR dissociation varies and should be monitored carefully. A prolonged dissociation time may lead to cell detachment.
5 Aspirate GCDR and add 1 ml TeSR‐E8 to each well of a 6‐well plate.
6 Detach cell colonies by scraping them with a cell scraper.
7 Transfer cell colonies into a 15‐ml tube and pipet up and down one to two times gently with a 1‐ml pipet.
  It is critical to break large pieces of colonies by pipetting to increase their adherence and survival after seeding.
8 Wash vitronectin‐coated plate with 1 ml dilution buffer for each well and add 2 ml TeSR‐E8.
9 Add 33 to 100 µl cell suspension from step 7 into each well (with a splitting ratio 1:10 to 1:30). Distribute cells evenly and place plate with the cells in an incubator at 37°C.
  It is important to ensure that the cell suspension is well mixed and the cells do not precipitate at the bottom of the tube. Gently tap the bottom of the tube before taking up the cell suspension from the tube.
10 Refresh cells every day starting 48 hr after passaging of human pluripotent stem cells (hPSCs).
  More than 2 ml TeSR‐E8 can be added when the cells become relatively dense 5 to 6 days after passaging of the hPSCs.
11 Passage cells once a week when hiPSC colonies start to contact each other (∼90% confluency).

### Passage hiPSCs for myeloid differentiation

Passage hiPSCs when they reach 90% confluency, as for the passaging for maintenance.

12 Prewarm a Matrigel‐coated 6‐well plate (or use a freshly prepared Matrigel‐coated plate) and TeSR‐E8 at room temperature for at least 30 min.
13 Dissociate hiPSC colonies, following steps 2 to 7.
14 Remove Matrigel and add 2 ml TeSR‐E8 in each well of the 6‐well plate.
15 Add 20 to 30 µl cell suspension into each well (with a split ratio 1:33 to 1:50). Distribute cells evenly and place in an incubator at 37°C.
  The seeding density of hiPSCs is of essential importance for differentiation efficiency. Too high a density will inhibit the emergence of hematopoietic cells from hemogenic endothelium from day 5 to day 9. When hiPSCs are dissociated from a confluent well, a higher split ratio (1:50) is recommended.

### Differentiate hiPSCs toward HPCs in 9 days

16 Mesoderm induction from day 0 to day 2: 24 hr after the passaging from step 15 (day 1), replace TeSR‐E8 medium with mesoderm induction medium (2 ml per well). Place plate with the cells into an incubator at 37°C.
  Optimal density of hiPSC colonies on day 0 should be around 30 colonies per well. Always prewarm medium to room temperature and add growth factors to the medium right before refreshing.
17 Hemogenic endothelium induction from day 2 to 5: On day 2, replace mesoderm induction medium with hemogenic endothelium induction medium (3 ml per well).
  On day 5, the differentiation efficiency of hemogenic endothelium can be determined by FACS analysis. More than 30% of the total cell population should be CD34+CD144+CD73‐.
18 Hematopoietic induction from day 5 to 9: On day 5, replace hemogenic endothelium induction medium with hematopoietic induction medium. Refresh cell culture on day 7 of differentiation. See Supporting Information Video 1 for the morphology change from day 7 to 9. Time lapse taken from differentiation day 7 to 9 at a frequency of 30 min each frame. The video plays at 10 frames/s. Scale bar represents 200 μm.
  On day 5, multiple cell layers should appear in the central area of the colonies while the edges stay as a monolayer. Most colonies have a dark sphere in the middle. Round single hematopoietic cells should start to appear in the central area already from day 7. At first, they will stay attached to the colonies, but as more cells appear, they will partly be released into the supernatant. On day 9, the differentiation efficiency of hematopoietic progenitors can be determined by FACS. More than 50% of CD43+ cells can be obtained on day 9.

### Monocyte induction from HPCs in 5 to 6 days

19 Prewarm Accutase solution to 37°C.
20 Gently detach loosely attached HPCs by flushing the colonies with the medium using a 5 ml‐pipet. Collect all floating cells and transfer cell suspension into a 50‐ml tube.
21 Wash each well with 1 ml Dulbecco's phosphate‐buffered saline (DPBS) and collect it into the same 50‐ml tube from step 20. Add 0.5 ml TrypLE to each well of a 6‐well plate and incubate at 37°C for 5 min. Tap plate several times and collect all cell suspension into the same 50‐ml tube from step 20.
22 Add 0.5 ml Accutase solution to each well of the 6‐well plate and incubate at 37°C for 5 min.
  The incubation time needed may vary for different hiPSC lines and seeding densities. Prolonged dissociation (up to 8 min) may be needed to detach most of the colonies. Some cells might remain attached.
23 Add 1 ml IF9S medium to each well. Scrape off cells using a cell scraper. Collect all cell suspension into the same 50‐ml tube from step 20.
  Avoid pipetting too vigorously and too often as it could damage the cells. Introducing too many dead cells and cell debris may be toxic and induce the activated phenotype of monocytes.
24 Add 1 ml IF9S medium to each well. Pipet up and down three to four times to wash off the remaining cells and collect whole cell suspension in the same 50‐ml tube from step 20.
25 Centrifuge cells collected in the 50‐ml tube at 1,100 rpm (300 × *g*) for 3 min at room temperature, then resuspend pellet in 12 ml monocyte induction medium for each 6‐well plate used to collect cells.
26 Distribute cell suspension over twelve wells of a 24‐well low‐attachment plate, adding 1 ml to each well.
  Mix cell suspension well before adding it to the 24‐well plate.
27 Refresh cells on day 12 with monocyte induction medium. To refresh, slowly flip plate 45° and remove 0.5 ml of the old medium from each well using a 5‐ml pipet, then add 1 ml fresh medium.
  Avoid disturbing the plate in order to keep all cells at the bottom before refreshing. Aspirate only upper supernatant from the well.
28 On day 14 or 15, collect all floating cells and perform isolation of CD14+ monocytes as described in Support Protocol 1.
  The time of harvesting can be either day 14 or 15 depending on each differentiation and hiPSC line. Cells should be collected when a small number of cells start to adhere and differentiate into macrophages. It is highly recommended that the differentiation efficiency is checked by FACS. Efficient monocyte induction should result in >50% of CD14+ cells.
  Sample data for Basic Protocol 1 are provided in Figure 1.

## Support Protocol 1 ISOLATION AND CRYOPRESERVATION OF MONOCYTES

This protocol describes methods for both isolation and cryopreservation of hiPSC‐mono, which have proven robust using at least three hiPSC lines (Cao et al., 2019). CD14 expression can be determined by FACS before monocyte isolation to determine the differentiation efficiency. The isolation should be performed on day 14 to day 15 depending on the differentiation efficiency and adherence of monocytes to the plate. The aim is to get the highest percentage of CD14+ cells before large numbers of adherent monocytes are observed at the time isolation is performed. Expected results are >50% CD14+ monocytes before isolation, with a yield of ∼5 × 10^6^ monocytes after isolation on day 14 or day 15 from each 6‐well plate of hiPSCs. More details on expected results are described in the Understanding Results section.

### Materials

Differentiated hiPSCs (see Basic Protocol 1)
CD14 MicroBeads, human (Miltenyi Biotec, cat. no. 130‐050‐201)
CryoStor® CS10 cryopreservation medium (Stemcell Technologies, cat. no. 07930)
FACS buffer (FACSB; see recipe)

Tubes, 15 ml (Greiner Bio‐One, cat. no. 188271)
Mr. Frosty™ Freezing Container
Sterile filters, 100 μm (CellTrics, cat. no. 04‐004‐2328)
Costar® 24‐well Clear Flat Bottom Ultra‐Low Attachment Multiple Well Plates (Corning, cat. no. 3473)
QuadroMACS Starting Kit (LS; Miltenyi, cat. no. 130‐091‐051)
Cryotubes (Greiner Bio‐One, cat. no. 123263)

1 Before collecting cells on day 14 or 15, prechill a Mr. Frosty™ Freezing Container to 4°C. Prewarm FACSB to room temperature.
2 Pipet cell suspension up and down twice using a 5‐ml pipet and collect the whole cell suspension in a 50‐ml tube.
3 Wash each well with 0.5 ml FACSB and collect it into the same tube. Spin down at 1,100 rpm (300 × *g*) for 3 min.
4 Discard supernatant and resuspend in 12 ml FACSB. Filter through a 100‐μm CellTrics® filter to obtain a single cell suspension. Collect all cells in a 15‐ml tube. Count total cell number and centrifuge at 1,100 rpm (300 × *g*) for 3 min at room temperature.
  It is critical to remove cell clumps by filtering, as they can block the column during magnetic isolation. Total cell number varies per differentiation. Usually 20 to 40 million cells can be collected from one 24‐well low‐attachment plate.
5 Discard supernatant and resuspend cells in FACSB (80 µl for every 10 million cells). Then add 50 µl CD14 MicroBeads for every 10 million cells. Mix well by flicking the bottom of the tube and incubate tube at 4°C for 15 min.
  Depending on the percentage of CD14+ cells (ranging from 40% to 80%) before isolation, 40 to 60 µl CD14 MicroBeads should be added for every 10 million cells.
6 Add 1.5 ml FACSB for every 10 million cells. Mix well by flicking the bottom of the tube and spin down at 1,100 rpm (300 × *g*) for 3 min at room temperature.
7 Discard supernatant and resuspend cells (up to 40 million) in 500 µl FACSB.
8 Assemble QuadroMACS Separator according to the manufacturer's instruction. Place LS column in the magnetic field. Wash LS column with 3 ml FACS buffer. Collect fluid into a 15‐ml tube.
  The QuadroMACS Separator is a very strong magnet and should be kept away from all electrical devices.
9 Wait until the reservoir of the column becomes empty, then add the 500‐µl cell suspension into the reservoir. Collect unbound cells into a 15‐ml tube.
10 Wait until the reservoir of the column becomes empty. Add 3 ml FACSB into the column to wash off the unbound cells. Collect unbound cells into a 15‐ml tube. Repeat washing for another two times (wash column three times in total).
11 Remove column from the magnetic field and put it on the top of a new 15‐ml tube. Add 5 ml FACS buffer to the column. Immediately flush out cells from the column by firmly pushing the plunger into the column.
12 Count number of cells in the collection tube. Take an aliquot for the characterization of purified monocytes.
  More than 90% of CD14+ monocytes can be obtained after the isolation step.
13 Spin down monocytes at 1,100 rpm (300 × *g*) for 3 min at room temperature.
14 Cryopreservation of monocytes: Resuspend monocytes in CryoStor® CS10 cryopreservation medium to get a final concentration of 3.75 million/ml. Aliquot 400 µl into each cryovial (1.5 million cells per vial).
  Monocytes and CS10 cryopreservation medium should be kept on ice. Cryovials should also be on ice during aliquoting of cells.
15 Place all cryovials into a Mr. Frosty™ Freezing Container and leave at −80°C for 24 hr. Then transfer all cryovials into liquid nitrogen for prolonged storage.
  Monocytes can be stored in liquid nitrogen for up to 2 years.

## Support Protocol 2 CHARACTERIZATION OF MONOCYTES

**Figure 2:**
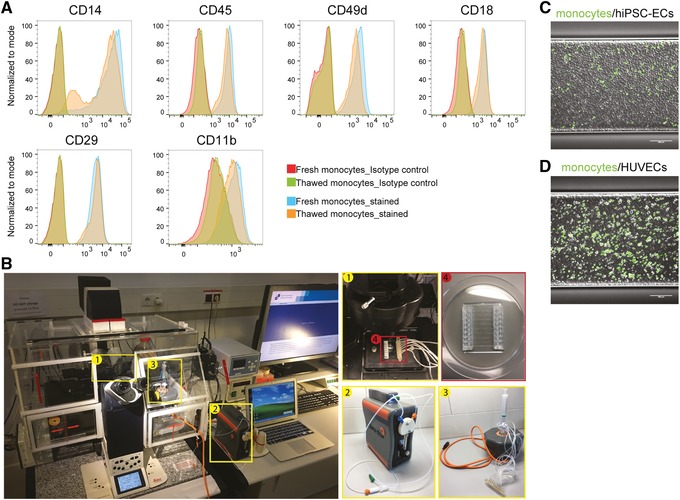
Functional characterization of hiPSC‐mono in a microfluidic adhesion assay. (**A**) FACS analysis of monocytes markers CD14, CD45, and integrins CD49d, CD18, CD29, and CD11b on freshly isolated and thawed monocytes. Isotype controls are shown in red. (**B**) Bench setup for the microfluidic adhesion assay, including an inverted fluorescent microscope with mounted live imaging chamber (5% CO_2_, 37°C, humidified), (1) microfluidic chip on the microscope stage, (2) a microfluidic pump, (3) an 8‐channel manifold, (4) an 8‐channel microfluidic chip. (**C**‐**D**) Representative images taken at the end of the flow assay with hiPSC‐ECs and HUVECs. Monocytes were labeled with DiOC6 (in green). Scale bar represents 200 μm. Abbreviations: hiPSC‐mono, hiPSC‐derived monocytes; FACS, fluorescence activated cell sorting; hiPSC‐ECs, hiPSC‐derived endothelial cells; HUVECs, human umbilical vein endothelial cells.

Isolated monocytes are characterized by Wright‐Giemsa staining (Fig. 1) or FACS (Fig. 2A). Wright‐Giemsa can be used to examine nucleus shape and cell size, and flow cytometry can be used to check for specific surface makers expressed on monocytes. Characterization can be done on either freshly isolated or cryopreserved monocytes after thawing. Cryopreserved hiPSC‐mono can be compared with freshly isolated monocytes for the expression of monocyte‐specific makers (Fig. 2A) as well as characterized functionally based on their ability to adhere to hiPSC‐derived endothelial cells (hiPSC‐ECs) or human umbilical vein endothelial cells (HUVECs; Fig. 2B‐D). The flow assay had been described in detail in our earlier publication (Halaidych, van den Hil, Mummery, & Orlova, 2018b) and is only briefly introduced here. In this assay, hiPSC‐mono should be highly adhesive to both hiPSC‐ECs and HUVECs that are stimulated with tumor necrosis factor alpha (TNF‐α; Fig. 2C and 2D). Primary blood monocytes (blood‐mono) can serve as controls for the characterization and functional assays of hiPSC‐mono.

### Materials

hiPSC‐derived monocytes (hiPSC‐mono; isolated from Support Protocol 1)
RPMI 1640 Medium (Gibco brand; Thermo Fisher Scientific, cat. no. 21875034)
10% FBS
Wright‐Giemsa Stain, Modified (MilliporeSigma, cat. no. WG16)
FACS buffer (FACSB; see recipe)
Fluorescent‐conjugated FACS antibodies

Tubes, 15 ml (Greiner Bio‐One, cat. no. 188271)
Round‐bottom tube, 5 ml FACS tube (BD Biosciences, cat. no. 352058)
White filter cards (Thermo Fisher Scientific, cat. no. 5991022)
Cytocentrifuge (Thermo Fisher Scientific, cat. no. A78300003)
Microscope slides (VWR, cat. no. 631‐1553)
MACSQuant® VYB Flow Cytometer (Miltenyi, cat. no. 130‐096‐116)

### Characterize hiPSC‐mono

#### By staining with Wright‐Giemsa

1a Centrifuge monocytes and resuspend in RPMI 1640 medium containing 10% FBS to reach a final concentration of 0.2 × 10^6^ cells/ml.
2a Label slides then mount them with the paper pad and cuvette in the metal holder of the cytocentrifuge.
  The protocol may need to be adjusted based on the model of cytocentrifuge used.
3a Load 100 to 200 μl monocyte suspension in each cuvette.
4a Spin at 800 rpm (273 × *g*) for 3 min at room temperature.
5a Disassemble each metal holder. Remove cuvette and paper pad carefully without disturbing cytocentrifuged cells. Label cell area with a permanent marker pen.
6a Dry slides at 37°C in an incubator dryer for 1 to 2 hr.
7a Add 1 ml Wright‐Giemsa stain solution to each slide to cover the cell area. After 30 s, add 1 ml deionized water and mix thoroughly with the dye by gently pipetting up and down with a 1‐ml pipet tip.
8a After 1 min, pick up the slide carefully with tweezers. Rinse slide thoroughly by putting the back side of the slide under the water tab for 1 min and air dry.
  Slides can be stored at room temperature for up to 3 years.

#### Analysis using flow cytometry

1b Place a 100‐μm CellTrics filter on the top of a 5‐ml FACS tube. Apply monocyte suspension through the filter and wash once with 2 ml FACSB.
2b Spin down at 1,100 rpm for 3 min at room temperature.
3b Wash cells once by resuspending them in 1 ml FACSB and spin down at 1,100 rpm for 3 min at room temperature.
4b Aspirate supernatant but leave around 50 μl inside. Add fluorescent‐conjugated FACS antibodies to the cell suspension to the desired working concentration. Suspend cells by flicking the bottom of the tube and incubate in the dark 30 min at 4°C.
  Antibodies used for the characterization of monocytes include those for CD45, CD14, CD11b, and CD18. Fc‐R blocking antibody should be added to each tube to reduce non‐specific binding of antibodies. Details of all antibodies are listed in the Reagents and Solutions section.
5b Turn off the light of the cell culture hood. Wash cell suspension with 1 ml FACSB and centrifuge at 1,100 rpm (300 × *g*) for 3 min at room temperature.
  Stained cells can be analyzed using flow cytometry immediately or fixed with 1% (w/v) paraformaldehyde (PFA) and analyzed the next day.
6b Analyze samples with a flow cytometer.
  We used the MACSQuant VYB flow cytometer with the following instrument settings: Blue/488 FITC, A488: 525/50; yellow/561 PE: 586/15; APC: 661/20; APC‐Cy7: 750LP. FlowJo software was used for data analysis.
  The MACSQuant VYB laser setup may require additional compensation between PE and APC channels. For that, extra compensation tubes need to be prepared and run before the analysis. Refer to the manual of the flow cytometer you are using for the setup and compensation procedures.
  Sample data of Support Protocol 2 are given in Figure 2.

## Basic Protocol 2 DIFFERENTIATION OF DIFFERENT SUBTYPES OF MACROPHAGES

This protocol describes methods for the induction of iPSDMs from freshly isolated or cryopreserved hiPSC‐mono, which had been tested and proven robust with multiple hiPSC lines previously (Cao et al., 2019). Cryopreserved hiPSC‐mono are thawed and seeded on FBS‐coated plates. An example of recovery is 43.2% ± 9.9%. Adherent monocytes are then further differentiated into M0 macrophages in the presence of M‐CSF for 4 days in defined IF9S medium. hiPSC‐mono (1.5 × 10^6^ cells; one cryovial) are seeded either into four or six wells of a 6‐well plate, in order to get a confluent monolayer of M0 macrophages in 4 and 6 days, respectively.

**Figure 3:**
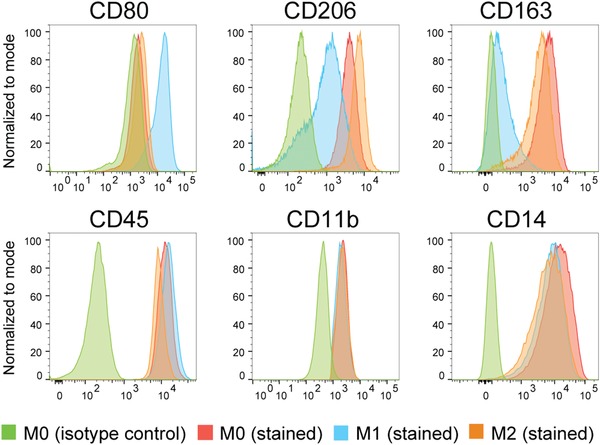
FACS analysis of macrophage subtype‐specific markers CD80 (M1), CD206 and CD163 (M2), and pan‐specific macrophage markers CD45, CD11b, and CD14 on iPSDMs. Abbreviations: FACS, fluorescence activated cell sorting; iPSDMs, hiPSC‐derived macrophages.

Macrophages are highly heterogeneous due to the diverse stimuli and immune response induced by local tissue environment; they can be classified as pro‐inflammatory M1 macrophages (M1) and anti‐inflammatory M2 macrophages (M2; Mantovani et al., 2004; Rőszer, 2015). In this protocol, we describe methods for the polarization of M1 using LPS and interferon γ (IFN‐γ) or M2 using interleukin 4 (IL‐4) for another 48 hr from M0 in IF9S medium. Identities of polarized macrophage subtypes can be examined based on the expression of macrophage‐specific markers by FACS (Fig. 3). Alternatively, a multiplex cytokine assay can be used to quantify their protein secretion profile. Expected results are ∼90% confluent monolayer of M0 macrophages 4 or 6 days after seeding. With this protocol, a confluent monolayer of polarized M1 and M2 iPSDMs can be derived from thawed hiPSC‐mono in 6 days, which could easily provide enough iPSDMs for multiple characterization and functional assays in vitro. More details on expected results are described in the Understanding Results section.

The major factor requiring optimization in this protocol is the seeding density of thawed hiPSC‐mono and the induction time of M0 macrophages. The quality of cryopreserved hiPSC‐mono may vary across different batches and hiPSC lines, which could affect their recovery rate and proliferation after thawing. In general, a confluent monolayer of M0 macrophages can still be obtained from a relatively low‐quality batch of cryopreserved hiPSC‐mono by a longer culture (up to 7 days) or a higher seeding density in M0 medium.

### Materials

Cryopreserved monocytes (from Support Protocol 1)
IF9S medium (see recipe)
M0 medium (see recipe)
M1 medium (see recipe)
M2 medium (see recipe)
FBS, South America, ultra‐low endotoxin (Biowest, cat. no. S1860)
Accutase‐Solution (PromoCell, cat. no. C‐41310)
TrypLE™ Express Enzyme (1×), no phenol red (Gibco brand; Thermo Fisher Scientific, cat. no. 12604021)

6‐well culture plates (Greiner Bio‐One, cat. no. 657160)
Tubes, 15 ml (Greiner Bio‐One, cat. no. 188271)
MACSQuant® VYB Flow Cytometer (Miltenyi, cat. no. 130‐096‐116)
Imaging plate, 96‐well (Corning, cat. no. 353219)

### Differentiation of monocytes into different subtypes of macrophages in 6 days

1 Coat four wells of a 6‐well plate with FBS and let stand overnight at 37°C (prepare one plate for each cryovial of monocytes). Prewarm IF9S medium to room temperature. Turn on the water bath and set to 37°C.
2 Take out cryovial with monocytes from liquid nitrogen and thaw in the water bath right away. Transfer all cell suspension in the cryovial into a 15‐ml tube that contains 10 ml IF9S medium. Wash cryovial once with medium to collect remaining cells.
  The thawing procedure should be performed as quickly as possible to minimize the time monocytes stay in cryopreservation medium. Move the cryovial in the water bath in a circle to thaw. Pipet cells gently to reduce mechanical stress.
3 Centrifuge the 15‐ml tube at 1,100 rpm (300 × *g*) for 3 min. Discard supernatant.
4 Resuspend monocytes from each cryovial in 8 ml M0 medium.
5 Remove FBS from the 6‐well plate and add 2 ml cell suspension into each well. Place plate back into the incubator and move plate in a cross‐like pattern to distribute cells evenly.
  Alternatively, cells can be seeded into six wells instead of four wells of a 6‐well plate so that they become confluent in 6 days, in order to harvest more M0 macrophages at the expense of two additional days of culture.
6 Refresh cells with M0 medium 2 days post thawing.
  Do not disturb cells during the first 2 days. Only a small number of cells adhere after 2 days (∼10%), which is normal and cells at this stage are still proliferating so that 80% confluency should be reached on day 4 (when starting with four wells of a 6‐well plate) or on day 6 (when starting with six wells of a 6‐well plate). An additional day may be needed for both starting formats to allow cells to become confluent before starting polarization.

### Polarization of iPSDMs

Polarization of M1 and M2 from M0 can be performed either in a 6‐well plate directly without dissociation or in a 96‐well plate for functional assays described in Support Protocol 4. For the polarization in a 96‐well plate, M0 are first dissociated and seeded in new 96‐well plates.

7 Two days before the assay, dissociate confluent M0 macrophages with Accutase solution at 37°C for 10 min.
8 Resuspend cells in M0 medium to reach 0.5 × 10^6^ cells/ml.
9 Seed 50 × 10^3^ cells (in 100 μl) into each well of a 96‐well black imaging plate.
  To obtain enough cells for analysis, seed two wells for each sample of the assay.
10 Start polarization 24 hr after seeding. Refresh cells with M0, M1, and M2 media, respectively, for polarization of M0, M1, and M2. Polarization lasts overnight (∼12 hr) for functional assays (in 96‐well format) and 48 hr for other analysis (in 6‐well format).
  It is critical to reach >80% confluency for M0 macrophages before polarization.

## Support Protocol 3 CHARACTERIZATION OF hiPSC‐DERIVED MACROPHAGES (iPSDMS)

iPSDMs that have been polarized can be characterized by flow cytometry and the multiplex cytokine assay. Flow cytometry can be used to determine specific surface marker expression on each macrophage subtype (Fig. 3). The multiplex cytokine assay is used to measure cytokine and chemokine secretion by macrophages. The major factor requiring optimization in this protocol is the dissociation time of polarized iPSDMs for FACS. In some cases, a longer dissociation time (10 to 20 min) may be required to detach iPSDMs from the plate. For the supernatant collected for the multiplex cytokine assay, we recommend diluting three times for the first trial and adjusting it later based on the result. Clear M1 and M2 specific signatures of polarized M1 and M2 macrophages should be observed by both FACS (Fig. 3) and multiplex cytokine assay (Cao et al., 2019).

### Materials

hiPSC‐derived macrophages (iPSDMs) subtypes (from Basic Protocol 2)
IF9S medium (see recipe)
Accutase‐Solution (PromoCell, cat. no. C‐41310)
FACS buffer (FACSB; see recipe)
FACSB‐10 (see recipe)

Tubes, 15 ml (Greiner Bio‐One, cat. no. 188271)
Round‐bottom tube, 5‐ml FACS tube (BD Biosciences, cat. no. 352058)
MACSQuant® VYB Flow Cytometer (Miltenyi, cat. no. 130‐096‐116)
LSR‐II flow cytometer (BD Biosciences)
Centrifuge 5810 R (Eppendorf)
96‐well round‐bottom plate (Greiner Bio‐One, cat. no. 651161)
LEGENDplex™ Human Inflammation Panel, 13‐plex (BioLegend, cat. no. 740809)
LEGENDplex™ Human Macrophage/Microglia Panel, 13‐plex (BioLegend, cat. no. 740503)

### iPSDMs analyses

#### By flow cytometric analysis

1a Prewarm Accutase solution to 37°C. Add 1 ml Accutase solution to each well of a 6‐well plate of iPSDMs at 37°C and let stand 10 min.
2a Add 1 ml FACSB‐10 to each well to stop the dissociation and wash off cells by pipetting three to four times using a P1000 pipet.
  Do not pipet more than four times as this may increase cell death. Check cells under the microscope after pipetting and continue with cell scraping if more than one‐third of cells did not come off the substrate.
3a Collect all of the cell suspension in a 50‐ml tube. Add 2 ml IF9S medium to each well and wash off cells by pipetting three to four times using a P1000 pipet.
  Check culture under the microscope after pipetting and if a large number of cells are still attached, scrape them off using a cell scraper.
4a Collect all the cell suspension into the same 50‐ml tube. Transfer an aliquot into a 5‐ml FACS tube for analysis. Centrifuge at 1,100 rpm (300 × *g*) for 3 min at room temperature.
5a Wash cells once with FACSB and spin down at 1,100 rpm for 3 min at room temperature.
6a Stain FACS antibodies and analyze by flow cytometry for monocytes as described in Support Protocol 2.

#### By multiplex cytokine assay

1b Collect supernatant of polarized macrophages. Centrifuge at 1,800 rpm (803 × *g*) for 5 min to remove debris.
  Cell culture supernatant can be aliquoted and stored at −20°C for up to 1 year. Avoid multiple (more than two) freeze‐thaw cycles.
2b The day before the assay, read the manual of the LEGENDplex^TM^ kit carefully. Design the plate layout of the assay. Prepare Wash Buffer and assay Standard Cocktail. Aliquot 80 μl Standard Cocktail into each Eppendorf tube and store at −80°C.
3b On the day of the assay, warm all reagents in the LEGENDplex^TM^ kit and the cell culture supernatant to room temperature.
4b Dilute supernatant three times in Assay Buffer. Load 15 μl standard or diluted supernatant into the 96‐well round‐bottom plate. Then, add 15 μl Assay Buffer and 15 μl mixed beads into each well; assays should be performed in duplicate.
  We added 15 μl of each component instead of 25 μl which was recommended in the kit instructions. This may save on the amount of samples and reagents used and does not affect the readout. However, <15 μl is not recommended.
5b Seal plate with a plate sealer and cover plate with aluminum foil. Shake plate at 800 rpm on a plate shaker 2 hr at room temperature.
6b Centrifuge plate at 1,050 rpm (219 × *g*) for 5 min. Remove plate seal and remove supernatant using a multichannel pipet. Do not disturb the blue pellet (beads) at the bottom.
7b Wash bead pellet with 200 μl Wash Buffer. Centrifuge again at 1,050 rpm (219 × *g*) and remove supernatant.
8b Add 15 μl Detection Antibodies to each well. Seal plate with the plate sealer and cover plate with aluminum foil. Shake plate at 800 rpm on a plate shaker 1 hr at room temperature.
9b Add 15 μl SA‐PE to each well. Seal plate with plate sealer and cover plate with aluminum foil. Shake plate at 800 rpm on a plate shaker 30 min at room temperature.
10b Centrifuge (1,050 rpm; 219 × *g*) and remove supernatant.
11b Add 150 μl Wash Buffer to each well and resuspend beads.
12b Analyze samples with flow cytometer.
  We used the LSR‐II (BD) with the following instrument settings: Blue laser: 575/26, Voltage: 360 V; Red laser: 660/20, Voltage: 400 V. Data was analyzed using LEGENDplex^TM^ data analysis software.
  Sample data of Support Protocol 3 are given in Figure 3.

## Support Protocol 4 FUNCTIONAL CHARACTERIZATION OF DIFFERENT SUBTYPES OF MACROPHAGES

**Figure 4:**
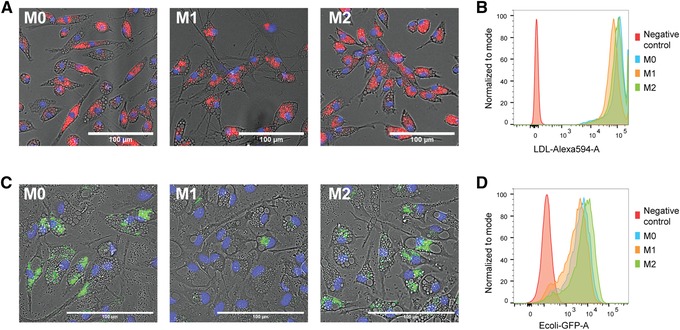
Endocytosis and phagocytosis of bacteria by iPSDMs. (**A**) Representative images of the AcLDL‐Alexa Fluor 594 uptake assay by different subtypes of iPSDMs. AcLDL positive uptake is shown in red; cell nuclei are stained with Hoechst in blue. Scale bar represents 100 μm. (**B**) FACS analysis of AcLDL‐Alexa Fluor 594 median fluorescence intensity of different macrophage subtypes. M0 macrophages only are included as negative control. (**C**) Representative images of bacterial phagocytosis by different subtypes of iPSDMs. Nuclei were stained with Hoechst in blue. GFP‐labeled (pHrodo green) *E. coli* were pH sensitive and only show green fluorescence inside macrophages. Scale bar represents 100 μm. (**D**) FACS analysis of *E. coli*‐GFP median fluorescence intensities in macrophage subtypes. M0 macrophages only are included as negative control. Abbreviations: iPSDMs, hiPSC‐derived macrophages; AcLDL, acetylated low‐density lipoprotein; FACS, fluorescence activated cell sorting; GFP, green fluorescent protein.

**Figure 5:**
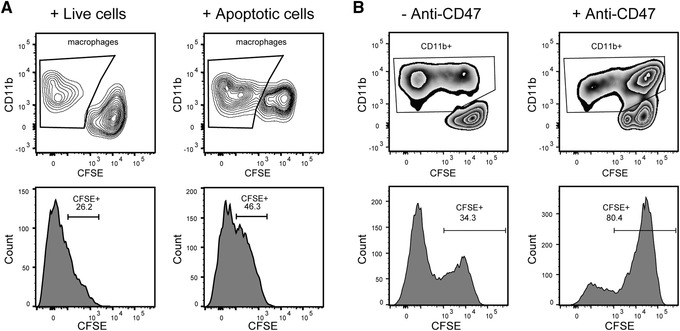
Efferocytosis and tumor phagocytosis assay of iPSDMs. (**A**) Efferocytosis assay of M0‐iPSDMs. Live cells (negative control) and apoptotic cells were labeled with CFSE and incubated with M0‐iPSDMs. CD11b+ macrophages are gated (upper panel) and their CFSE intensity is shown in histograms (lower panel). (**B**) Phagocytosis of Jurkat cells by M0‐iPSDMs. CFSE‐labeled Jurkat cells were incubated with or without anti‐CD47 blocking antibody and incubated with macrophages for 2 hr. CD11b+ macrophages are gated (upper panel) and their CFSE intensity is shown in histograms (lower panel). Abbreviations: iPSDMs, hiPSC‐derived macrophages; CFSE, carboxyfluorescein succinimidyl ester.

This protocol is designed to establish different assays for the functional characterization of iPSDMs, including Dil‐acetylated low‐density lipoprotein (AcLDL) uptake assay (Fig. 4A and 4B), bacterial phagocytosis assay (Fig. 4C and 4D), and efferocytosis assay, to examine their ability to clear up apoptotic cells (Fig. 5A) or the tumor phagocytosis assay to examine tumoricidal activity (Fig. 5B). These assays could be used to check the quality of iPSDMs produced with this and other published differentiation protocols. Similarities and differences could be observed when PBDMs and iPSDMs were compared using different assays as we previously observed (Cao et al., 2019).

### Materials

hiPSC‐derived macrophage (iPSDM) subtypes (from Basic Protocol 2)
IF9S medium (see recipe)
Lipid‐free IF9S medium (see recipe)
PS‐free IF9S medium (see recipe)
Jurkat medium (see recipe)
Accutase‐Solution (PromoCell, cat. no. C‐41310)
Alexa Fluor™ 594 AcLDL (Invitrogen brand, Thermo Fisher Scientific, cat. no. L35353)
NucBlue™ Live ReadyProbes™ Reagent (Invitrogen brand, Thermo Fisher Scientific, cat. no. R37605)
FACSB (see recipe)
pHrodo™ Green *E. coli* BioParticles™ Conjugate for Phagocytosis (Invitrogen brand, Thermo Fisher Scientific, cat. no. P35366)
4% paraformaldehyde (PFA; see recipe)
Dulbecco's phosphate‐buffered saline (DPBS)
TeSR‐E8 medium
FACSB‐10 (see recipe)
CellTrace™ CFSE Cell Proliferation Kit, for flow cytometry (Invitrogen brand; Thermo Fisher Scientific, cat. no. C34554)
Jurkat tumor cells (provided by Dr. Luuk Hawinkels, Leiden University Medical Center, LUMC)
Anti‐human CD11b, Vioblue conjugated (Miltenyi Biotec, cat. no. 130‐097‐336)
Anti‐human CD47, 1:200 (Bio‐rad, cat. no. MCA911)
Annexin V, Pacific Blue™ conjugate, for flow cytometry (Invitrogen brand; Thermo Fisher Scientific, cat. no. A35122)
Annexin Binding Buffer (5×), for flow cytometry (Invitrogen brand; Thermo Fisher Scientific, cat. no. V13246)
Propidium Iodide (PI) Solution (Miltenyi Biotec, cat. no. 130‐093‐233)

6‐well culture plates (Greiner Bio‐One, cat. no. 657160)
96‐well imaging plate (Corning, cat. no. 353219)
T75 flask
5‐ml FACS tube
MACSQuant® VYB Flow Cytometer (Miltenyi, cat. no. 130‐096‐116)
UV lamp

### Functional characterization of iPSDMs

#### AcLDL uptake assay

For the AcLDL assay, it is essential to use lipid‐free IF9S medium to deplete low‐density lipoprotein (LDL) during the polarization of iPSDMs.

1a On the day of the assay, dilute Alexa Fluor 594 AcLDL in lipid‐free IF9S medium to a final concentration of 5 μg/ml (1 μl in 199 μl, 1/200 dilution). Add 100 μl to each well of macrophages and incubate at 37°C for 4 hr. Leave two wells without AcLDL as a negative control.
2a Wash cells once with 100 μl lipid‐free IF9S medium.
3a Prepare NucBlue solution by adding two drops of NucBlue™ Live ReadyProbes™ reagent into 1 ml lipid‐free IF9S medium. Add 100 μl to each well of macrophages and incubate at 37°C for 20 min.
4a Optionally, take images with the microscope during the incubation of NucBlue. Set incubation chamber of the microscope to 37°C and 5% CO_2_.
5a Remove NucBlue solution and dissociate macrophages with Accutase 10 min at 37°C.
6a Collect cells from duplicate wells in a 5‐ml FACS tube. Wash once with FACSB and analyze with a flow cytometer right away to measure Alexa Fluor 594 intensity in cells.

#### Bacterial phagocytosis assay

The bacterial phagocytosis assay should be performed in a molecular biology lab and not in the cell culture room to avoid bacterial contamination of cultured cells (all reagents, cells, and equipment should be kept out of the cell culture lab).

1b On the day of the assay, take one vial of pHrodo Green *E. coli* BioParticles conjugate for all 30 wells to be tested. Add 1 ml PS‐free IF9S medium. Vortex 30 s and transfer suspension into a clean glass tube. Add another 2 ml PS‐free IF9S medium and incubate 30 min at room temperature.
2b Sonicate pHrodo Green *E. coli* BioParticles in PS‐free IF9S medium 15 min and incubate 30 min at room temperature.
3b Vortex pHrodo Green *E. coli* BioParticles in PS‐free IF9S medium 30 s and transfer to a 15‐ml tube. Centrifuge at 200 rpm (3.72 × *g*) for 1 min at room temperature. Transfer supernatant to a new tube.
  It is crucial to obtain a homogenous single cell suspension. Cell clumps can be avoided by low‐speed centrifugation. Take a small aliquot diluted in medium and check under a microscope to make sure all clumps are depleted.
4b Add 70 μl pHrodo Green *E. coli* BioParticles (supernatant from step 3b) per well of a 96‐well plate containing macrophages (from Basic Protocol 2). Incubate 30 min at 37°C.
5b Prepare NucBlue solution and add 100 μl to each well. Incubate at 37°C for 20 min.
6b Optionally, take images with the microscope during the incubation of NucBlue. Set incubation chamber of the microscope to 37°C and 5% CO_2_.
7b Remove NucBlue solution and dissociate macrophages with Accutase solution 10 min at 37°C.
8b Collect cells from duplicate wells into a 5‐ml FACS tube. Wash once with FACSB. Fix cells with 4% PFA and wash once with DPBS.
  It is important to fix the cells in order to kill bacteria and avoid contamination of other reagents and equipment. Fixed cells can be stored in DPBS in the dark for up to 48 hr at 4°C.
9b Analyze with a flow cytometer to measure Alexa Fluor 488 intensity in cells.

#### Efferocytosis assay

This assay can be used to examine the capability of iPSDMs for taking up apoptotic cells. Live hiPSCs are also needed in this assay as a negative control.

1c Culture hiPSCs in a normal manner as described in Basic Protocol 1, as a source of apoptotic cells for the assay.
  To obtain enough hiPSCs, perform the assay when hiPSCs are confluent. Usually one well of confluent hiPSCs in a 6‐well plate are sufficient for 15 to 20 wells of the assay in a 96‐well plate format. Prepare one extra well of hiPSCs for live cell control (non‐apoptotic cells).
2c On the day of the assay, dissociate hiPSCs with Accutase solution at 37°C for 5 min. Then add 2 ml TeSR‐E8 medium and pipet three to four times with a P1000 pipet to obtain a single cell suspension. Centrifuge at 1,100 rpm (300 × *g*) for 3 min at room temperature.
3c Spin down hiPSCs and resuspend in 3 ml TeSR‐E8. Transfer cell suspension to a 35‐ × 10‐mm dish.
4c Place dish under the UV lamp at ∼3‐cm distance. Set light intensity to 35 J/cm^2^. Remove lid and expose cells to UV light for 5 min.
  It is necessary to optimize the UV light intensity and exposure time depending on the UV lamp used. Cells are more sensitive to the change of exposure time rather than the change of UV light intensity. Be aware that UV light exposure may cause eye and skin damage. Perform the experiment in an enclosed space or wear protective mask and clothes.
5c Place dish with cells back in the incubator for 1 hr. During this incubation time, start to dissociate another well of hiPSCs using Accutase solution for the negative control without UV treatment.
6c Spin down both apoptotic (UV treated) and live hiPSCs at 1,100 rpm (300 × *g*) for 3 min.
  Take an aliquot of both apoptotic and live hiPSCs to determine the percentage of apoptotic cells. Apoptotic cells should be Annexin V+ and PI‐. More than half of hiPSCs should be apoptotic due to the UV treatment.
7c Add 2 μl carboxyfluorescein succinimidyl ester (CFSE) dye stock solution (see recipe) into 2 ml PBS to get a 5 μM CFSE working solution. Resuspend both tubes of hiPSCs in 1 ml CFSE solution. Incubate at 37°C for 20 min.
8c Add 5 ml FACSB‐10 to each tube of hiPSCs and count cell number of each tube. Centrifuge at 1,100 rpm (300 × *g*) for for 3 min.
9c Resuspend cells in IF9S medium to a final cell concentration of 4 million/ml.
10c Add 50 μl cell suspension to each well of iPSDMs. Incubate at 37°C for 1 hr.
11c Wash each well of iPSDMs with 100 μl IF9S medium.
12c Dissociate iPSDMs with Accutase at 37°C for 10 min. Collect cells from duplicate wells in a 5‐ml FACS tube. Wash once with FACSB.
13c Stain cells with anti‐CD11b fluorescent‐conjugated antibody 30 min at 4°C.
14c Wash once with FACSB and analyze by flow cytometry immediately to measure the CFSE intensity within the population of CD11b+ iPSDMs.

#### Tumor phagocytosis assay

Jurkat cells (an immortalized line of human T‐lymphocyte cells) are used in this assay as targeted tumor cells to be phagocytosed by iPSDMs. Jurkat cells without anti‐CD47 antibody treatment serve as a negative control. Jurkat cells are cultured in Jurkat medium in a T75 flask and passaged every 4 days at a 1:10 ratio.

1d On the day of the assay, collect Jurkat cells and count cell number.
2d Calculate volume of Jurkat cell suspension needed (200,000 cells per well). Centrifuge at 1,100 rpm (300 × *g*) for 3 min.
3d Resuspend cells in 1 ml CFSE working solution (5 μM in PBS, 1 μl in 999 μl, 1/1000 dilution). Incubate at 37°C, 20 min.
4d Add 5 ml FACSB‐10 to the tube. Aliquot cell suspension into two tubes: One for the negative control without anti‐CD47 treatment and the other for the experimental group with anti‐CD47 treatment. Centrifuge both tubes at 1,100 rpm (300 × *g*) for 3 min.
5d Suspend cells in both tubes in 50 μl FACSB. Add 3 μl anti‐CD47 (3 μg) for every million Jurkat cells in the experimental tube. Leave the negative control tube untreated. Incubate at 4°C, 30 min.
6d Wash once with FACSB and resuspend cells in both tubes in IF9S medium to a final concentration of 4 million/ml.
7d Add 50 μl cell suspension to each well of iPSDMs. Incubate at 37°C, 2 hr.
8d Wash each well of iPSDMs with 100 μl IF9S medium.
9d Dissociate iPSDMs with Accutase at 37°C, 10 min. Collect cells from duplicate wells into a 5‐ml FACS tube. Wash once with FACSB.
10d Stain cells with anti‐CD11b fluorescent‐conjugated antibody 30 min at 4°C.
11d Wash once with FACSB and analyze by flow cytometry right away to measure CFSE intensity within CD11b+ iPSDMs.
  Sample data of Support Protocol 1 are presented in Figures 4 and 5.

## REAGENTS AND SOLUTIONS

### Activin A stock solution, 25 µg ml^−1^

Reconstitute at a concentration of 25 µg ml^−1^ in PBS containing 1% (w/v) BSA. Prepare aliquots and store for up to 1 year at −80°C.

### l‐Ascorbic acid 2‐phosphate (AA2P), 5 mg ml^−1^

Add 250 mg AA2P to 50 ml of distilled water, non‐sterile.

Store at −20°C for up to 1 year.

### Basic fibroblast growth factor (FGF2) stock solution, 100 µg ml^−1^

Reconstitute at 100 μg ml^–1^ in PBS containing 0.1% (w/v) BSA. Prepare aliquots and store at –80°C for up to 1 year. Avoid repeated freeze‐thaw cycles.

### Bone morphogenetic protein 4 (BMP4) stock solution, 25 µg ml^−1^

Reconstitute at a concentration of 100 µg ml^−1^ in 4 mM HCl containing 1% (w/v) BSA. Then dilute further to 25 µg ml^−1^ in PBS containing 0.1% (w/v) BSA (see recipe). Prepare aliquots and store for up to 1 year at −80°C.

### BSA, 1% (w/v) in PBS

Dissolve 0.5 g BSA in 50 ml PBS. Sterilize solution by filtration using a 0.22‐μm membrane filter. Store for up to 4 weeks at 4°C.

### Carboxyfluorescein succinimidyl ester (CFSE) dye stock solution

Take one vial CFSE dye included in the CellTrace™ CFSE cell proliferation kit and reconstitute in 18 μl DMSO (provided in the kit) to prepare a 5 mM CFSE stock solution.

Store protected from light at −20°C for up to 1 year.

### CHIR99021 (CHIR) solution, 4 mM

Reconstitute 10 mg in 5.37 ml in DMSO. Prepare aliquots and store at −20°C for up to 1 year.

### EDTA, 0.5 M (pH 8.0)

Add 186.1 g EDTA to 800 ml distilled water. Adjust pH to 8.0 with NaOH and add distilled water to a final volume of 1 L. Filter solution through a 0.22‐μm membrane; filter and sterilize by autoclaving. Store for up to 1 year at room temperature (20°C).

### Fluorescence activated cell sorting buffer (FACSB)

Dissolve 1.25 g BSA in 250 ml PBS and add 1 ml 0.5 M EDTA (pH 8.0; see recipe). Sterilize by using a Stericup filter (0.22 μm).

Store for up to 4 weeks at 4°C.

### Fluorescence activated cell sorting buffer (FACSB)‐10

Add 5 ml FBS to 45 ml FACSB (see recipe). Sterilize by filtration using a 0.22‐μm membrane filter. Store for 4 weeks at 4°C.

Concentration: 10% (v/v) FBS.

### Hematopoietic induction medium

Prepare hematopoietic induction medium using the volumes listed below. Always prepare fresh.

**Table:** 

Composition	Volume	Final concentration
IF9S (see recipe)	25 ml	
IL‐6 (100 µg ml^−1^; see recipe)	12.5 µl	50 ng ml^−1^
IL‐3 (20 µg ml^−1^; see recipe)	12.5 µl	10 ng ml^−1^
TPO (50 µg ml^−1^; see recipe)	25 µl	50 ng ml^−1^
FGF2 (100 µg ml^−1^; see recipe)	12.5 µl	50 ng ml^−1^
SCF (50 µg ml^−1^; see recipe)	25 µl	50 ng ml^−1^
VEGF (50 µg ml^−1^; see recipe)	25 µl	50 ng ml^−1^

### Hemogenic endothelium induction medium

Prepare hemogenic endothelium induction medium using the volumes listed below. Always prepare fresh.

**Table:** 

Composition	Volume	Final concentration
IF9S (see recipe)	25 ml	
VEGF (50 µg ml^−1^; see recipe)	25 µl	50 ng ml^−1^
SB431542 (20 nM; see recipe)	12.5 µl	10 µM
FGF2 (100 µg ml^−1^; see recipe)	12.5 µl	50 ng ml^−1^
SCF (50 µg ml^−1^; see recipe)	25 µl	50 ng ml^−1^

### IF9S medium

Prepare 250 ml IF9S medium using the volumes listed below. Sterilize medium using a Stericup filter and store at 4°C for up to 3 weeks.

The formulation is based on previously described IF9S medium (Uenishi et al., 2014).

**Table:** 

Composition	Volume	Final concentration
IMDM	117.25 ml	
Ham's F‐12 nutrient mixture (F12; see recipe)	117.25 ml	
PVA (5%; see recipe)	50 µl	10 mg ml^−1^
lipids (100×; see recipe)	250 µl	0.1%
ITS‐X (100×; see recipe)	5 ml	2%
αMTG (1.3%; see recipe)	750 µl	40 µl L^−1^
AA2P (5 mg ml^−1^; see recipe)	3.2 ml	64 mg L^−1^
Glutamax (200 mM)	2.5 ml	2 mM
non‐essential amino acid (NEAA) solution (100×)	2.5 ml	1%
Pen/Strep (5000 U ml^−1^)	1.25 ml	0.5%

### Interferon γ (IFN‐γ) stock solution, 40 µg ml^−1^

Reconstitute at 40 μg ml^–1^ in PBS containing 0.1% (w/v) BSA. Prepare aliquots and store at –20°C or below for up to 1 year. Avoid repeated freeze‐thaw cycles.

### Interleukin 3 (IL‐3) stock solution, 20 µg ml^−1^

Reconstitute at 20 μg ml^–1^ in PBS containing 0.1% (w/v) BSA. Prepare aliquots and store at –20°C or below for up to 1 year. Avoid repeated freeze‐thaw cycles.

### Interleukin 4 (IL‐4) stock solution, 20 µg ml^−1^

Reconstitute at 20 μg ml^–1^ in PBS containing 0.1% (w/v) BSA. Prepare aliquots and store at –20°C or below for up to 1 year. Avoid repeated freeze‐thaw cycles.

### Interleukin 6 (IL‐6) stock solution, 100 µg ml^−1^

Reconstitute at 100 μg ml^–1^ in PBS containing 0.1% (w/v) BSA. Prepare aliquots and store at –20°C or below for up to 1 year. Avoid repeated freeze‐thaw cycles.

### Jurkat medium

Prepare using the volumes listed below. Sterilize using a Stericup filter and store for up to 3 weeks at 4°C.

**Table:** 

Composition	Volume	Final concentration
RPMI 1640	250 ml	
FBS	25 ml	10%
Glutamax (200 mM)	2.5 ml	2 mM
2‐mercaptoethanol (BME; 50 mM)	250 µl	50 µM
Pen/Strep (5000 U ml^−1^)	2.5 ml	1%

### Lipid‐free IF9S medium

Prepare IF9S medium (see recipe) without lipids (100×).

Store at 4°C for up to 3 weeks.

### Lipopolysaccharide (LPS) stock solution, 100 µg ml^−1^

Reconstitute at 100 μg ml^–1^ in PBS containing 0.1% (w/v) BSA. Prepare aliquots and store at –20°C or below for up to 1 year. Avoid repeated freeze‐thaw cycles.

### M0 medium

Prepare using the volumes listed below. Always prepare fresh.

**Table:** 

Composition	Volume	Final concentration
IF9S (see recipe)	25 ml	
M‐CSF (80 µg ml^−1^; see recipe)	25 µl	80 ng ml^−1^

### M1 medium

Prepare using the volumes listed below. Always prepare fresh.

**Table:** 

Composition	Volume	Final concentration
IF9S (see recipe)	25 ml	
LPS (100 µg ml^−1^; see recipe)	2.5 µl	10 ng ml^−1^
IFN‐γ (40 µg ml^−1^; see recipe)	12.5 µl	20 ng ml^−1^

### M2 medium

Prepare using the volumes listed below. Always prepare fresh.

**Table:** 

Composition	Volume	Final concentration
IF9S (see recipe)	25 ml	
IL‐4 (20 µg ml^−1^; see recipe)	25 µl	20 ng ml^−1^

### Macrophage colony‐stimulating factor (M‐CSF) stock solution, 80 µg ml^−1^

Reconstitute at 80 μg ml^–1^ in PBS containing 0.1% (w/v) BSA. Prepare aliquots and store at –20°C or below for up to 1 year. Avoid repeated freeze‐thaw cycles.

### Matrigel‐coated 6‐well plate

Thaw 100 µl Matrigel on ice for each 6‐well cell culture plate. Aliquot 12 ml cold (4°C) Dulbecco's modified Eagle's medium/nutrient mixture F‐12 (DMEM/F12) medium into a 50‐ml tube. Cool a pipet tip by pipetting up and down the cold DMEM/F12 medium several times and use it to transfer thawed Matrigel into medium. Mix with a cold (4°C) pipet and add 2 ml to each well. Leave plate at room temperature for 1 hr.

Use the plate right away or seal with Parafilm and store at 4°C for up to 2 weeks.

### Mesoderm induction medium

Prepare mesoderm induction medium using the volumes listed below. Always prepare fresh.

**Table:** 

Composition	Volume	Final concentration
IF9S (see recipe)	25 ml	
Activin A (25 µg ml^−1^; see recipe)	15 µl	15 ng ml^−1^
BMP4 (25 µg ml^−1^; see recipe)	40 µl	40 ng ml^−1^
CHIR (4 mM; see recipe)	9.4 µl	1.5 µM

### Monocyte induction medium

Prepare monocyte induction medium using the volumes listed below. Always prepare fresh.

**Table:** 

Composition	Volume	Final concentration
IF9S (see recipe)	25 ml	
M‐CSF (80 µg ml^−1^; see recipe)	25 µl	80 ng ml^−1^
IL‐6 (100 µg ml^−1^; see recipe)	12.5 µl	50 ng ml^−1^
IL‐3 (20 µg ml^−1^; see recipe)	12.5 µl	10 ng ml^−1^

### α‐Monothioglycerol (αMTG) solution, 1.3% (v/v)

Add 130 µl αMTG to 9.87 ml Iscove's modified Dulbecco's medium (IMDM) and store at 4°C protected from light for up to 1 year.

### Paraformaldehyde (PFA) solution, 4% (w/v)

Add 1 volume of PFA (8%, w/v; see recipe) to 1 volume of 0.2 M phosphate buffer (pH 7.4; see recipe). Cover mixture with foil and store at 4°C for up to 2 weeks.

### Paraformaldehyde (PFA) solution, 8% (w/v)

Add 40 g PFA to 400 ml Milli‐Q water. Heat PFA to 60°C and stir at medium speed. After a few minutes, add ∼10 drops of 1 N NaOH to dissolve PFA granules. After 1 to 2 hr, the solution will become translucent. Let solution cool down and add Milli‐Q water to a total volume of 500 ml. Store at 4°C for up to 2 months.

### Phosphate buffer, 0.2 M (pH 7.4)

Prepare solution 1: Dissolve 8.28 g NaH_2_PO_4_·H_2_O in 300 ml Milli‐Q water.
Prepare solution 2: Dissolve 10.78 g Na_2_HPO_4_·2H_2_O in 300 ml Milli‐Q water.
Add solution 1 to solution 2 until a pH of 7.4 is obtained, to make 0.2 M phosphate buffer (pH 7.4). Store at room temperature indefinitely (no expiration date).

### Poly(vinyl alcohol) (PVA), 5% (w/v)

Add 2 g PVA to 40 ml of distilled water in a 50‐ml tube. Leave tube for 2 days on a roller bank at room temperature. Heat tube 10 min at 75°C to completely dissolve PVA. Prepare aliquots and store at 4°C (non‐sterile) for up to 1 year.

### PS‐free IF9S medium

Prepare IF9S medium (see recipe) without Pen/Strep (5000 U ml^−1^).

Store at 4°C for up to 3 weeks.

### SB431542 solution, 20 mM

Reconstitute 10 mg in 1.3 ml DMSO. Prepare aliquots and store at −20°C for up to 1 year.

### Stem cell factor (SCF) stock solution, 50 µg ml^−1^

Reconstitute at 50 μg ml^–1^ in PBS containing 0.1% (w/v) BSA. Prepare aliquots and store at –20°C or below for up to 1 year. Avoid repeated freeze‐thaw cycles.

### Thrombopoietin (TPO) stock solution, 50 µg ml^−1^

Reconstitute at 50 μg ml^–1^ in PBS containing 0.1% (w/v) BSA. Prepare aliquots and store at –20°C or below for up to 1 year. Avoid repeated freeze‐thaw cycles.

### Transforming growth factor beta‐3 (TGFβ3) solution, 5 µg ml^−1^

Reconstitute contents of the vial to a concentration of 5 µg ml^−1^ in 4 mM HCl containing 0.1% (w/v) BSA. Prepare aliquots and store up to 1 year at −80°C.

### TX‐100 in PBS, 0.1% (v/v)

Add 50 µl TX‐100 to 50 ml PBS. Sterilize solution by filtration using a 0.22‐μm membrane filter. Store for up to 1 year at room temperature.

### Vascular endothelial growth factor (VEGF) stock solution, 50 µg ml^−1^

Reconstitute at 50 μg ml^–1^ in PBS containing 0.1% (w/v) BSA. Prepare aliquots and store for up to 1 year at −80°C.

### Vitronectin‐coated 6‐well plate

Warm up CellAdhere™ dilution buffer (Stemcell Technologies, cat. no. 07183) to room temperature. Thaw 80 µl Vitronectin XF™ (Stemcell Technologies) at room temperature and add to 2.42 ml dilution buffer. Mix well and add 1.25 ml to each well of 6‐well cell suspension plate. Distribute vitronectin to cover the whole well and incubate at room temperature 1 hr.

Use the plate right away or seal with Parafilm and store at 4°C for up to 2 weeks.

## COMMENTARY

### Background Information

During embryo hematopoiesis, HPCs are generated from HEs in the aorta‐gonad‐mesonephros (AGM) region of the embryo which forms from the mesoderm lineage. Several protocols have been reported for the induction of monocytes and macrophages from hPSCs that rely on mimicking the hematopoiesis in vivo. In 2009, Choi et al. successfully differentiated hiPSCs into CD43+CD45+ hematopoietic progenitors through the coculture with OP9 stromal cells; these could later be differentiated into mature macrophages using M‐CSF and IL‐1β (Choi, Vodyanik, & Slukvin, 2009; 2011). In 2008, an embryoid body (EB)‐based differentiation method was reported to induce monocyte‐like cells from hPSCs using M‐CSF and IL‐3 (Karlsson et al., 2008; Wilgenburg, Browne, Vowles, & Cowley, 2013). In 2015, a similar EB‐based method was described, which can be used to derive granulocytes, monocytes, and macrophages from hPSCs (Lachmann et al., 2015). Recently, this continuous harvesting method was successfully translated into bioreactors for mass production of iPSDMs (Ackermann et al., 2018). Also, in 2015, Zhang et al. published another EB‐based protocol which used single‐time point harvesting instead of continuous harvesting. They induced mesoderm, HPCs, and myeloid cells from hiPSCs sequentially, although the presence of HEs was not examined in their differentiation system (Zhang et al., 2015).

More recently, monolayer differentiation protocols have been reported by several groups. Uenishi et al. established an elegant hematopoietic induction method from hPSCs using a stepwise strategy. Mesoderm cells were first induced with BMP4, Activin A, lithium chloride (LiCl), and FGF2, which were further differentiated into HEs using VEGF and FGF2. Then HPCs were induced from HEs with SCF, IL‐6, IL‐3, TPO, FGF2, and VEGF, which showed multilineage differentiation potential toward lymphoid and myeloid cells (Uenishi et al., 2014). In 2017, Takata et al. reported a comparable stepwise method to derive primitive macrophages from hiPSCs (Takata et al., 2017). Hypoxia conditions were utilized for hematopoietic induction in both monolayer protocols.

Our protocol described here utilizes the monolayer differentiation approach rather than EBs. It has the particular advantage that it is more efficient and robust than EB‐based methods. For the induction of HPCs from hiPSCs (day 0 to 9), our method is similar to that of Uenishi et al. (2014), although different growth factors were used for the induction of mesoderm and HEs (Cao et al., 2019). Mesoderm cells are first induced from hiPSCs in 2 days and then into CD144+CD73‐ HEs for another 3 days, using our previously established endothelial cell (EC) induction method (Orlova et al., 2014a; 2014b). To induce CD43+ HPCs from HEs, SCF, IL‐6, IL‐3, TPO, FGF2, and VEGF are applied from day 5 to day 9, much like the previously published protocol by Uenishi et al. (2014) with the exception that we use normoxic conditions. Next, we induced the formation of CD14+ monocytes from HPCs. All cells were dissociated on day 9 and cultured in the presence of IL‐3, IL‐6, and M‐CSF in suspension. After 5 to 6 days culture, more than half of the cells became CD14+ monocytes which could be purified and cryopreserved and later thawed for functional analysis or further polarization toward different macrophage subtypes. A unique feature of this protocol compared to other differentiation methods is the dissociation step for HPCs on day 9, followed by suspension culture which notably maximizes differentiation efficiency compared with collecting only the floating cells continuously (Karlsson et al., 2008; Lachmann et al., 2015) or at a single‐time point (Takata et al., 2017; Wilgenburg et al., 2013; Zhang et al., 2015).

Compared to continuous harvesting, single‐time point harvesting has a relatively lower total cell yield but with higher reproducibility across different lines and batches which is critical for disease modeling. hiPSC‐mono from this protocol could be cryopreserved and still preserve their phenotype (Fig. 2A) and functionalities (Fig. 2C and 2D), which greatly facilitates their application in disease modeling in vitro. However, this protocol is not ideal for mass production of cells within bioreactors compared to continuous harvesting methods. Another limitation of this protocol is the isolation step for CD14+ monocytes using magnetic beads, which is relatively labor intensive and expensive. Lastly, although defined and xeno‐free medium is used throughout the protocol, the extracellular matrix used for coating is of animal origin and includes Matrigel and FBS. This may introduce batch‐to‐batch variations and needs to be optimized or defined for application in regenerative medicine or clinical studies.

Functionality of hiPSC‐mono can be examined via their adhesion to immobilized recombinant proteins that mediate the leukocyte recruitment cascade during inflammation, such as fibronectin, E‐selectin, intercellular adhesion molecule‐1 (ICAM‐1), and vascular cell adhesion molecule‐1 (VCAM‐1). Alternatively, adhesion to activated, TNF‐α, lipopolysaccharide (LPS), or interleukin 1β (IL‐1β) stimulated endothelial cells (ECs) can be used. Traditionally, static assays are used; however, they lack the physiological flow that is important for leukocyte adhesion and is essential in shear‐induced activation of leukocyte integrins. Therefore, leukocyte adhesion in a microfluidic flow assay is a more physiologically relevant way of assessing functionality of hiPSC‐mono and allows cross comparison of them with primary cells. Previously we described a detailed protocol to study adhesion of monocytes to hiPSC‐ECs in a microfluidic chip (Halaidych et al., 2018b). The protocol was applied to study adhesion of human monocytic cell line (THP1; Halaidych et al., 2018a), blood‐mono, and hiPSC‐mono (Cao et al., 2019). We have previously shown that hiPSC‐ECs when compared to primary HUVECs exhibit lower induction of leukocyte pro‐adhesive receptors, such as E‐selectin and ICAM‐1, and lack upregulation of VCAM‐1 upon treatments with various pro‐inflammatory cytokines (TNF‐α, LPS, and IL‐1β; Halaidych et al., 2018a). Pre‐conditioning of hiPSC‐ECs with bone morphogenetic protein 9 (BMP9) enhances inflammatory responses and results in an increase in VCAM‐1 expression (Cao et al., 2019), which is an important receptor for very late antigen 4 (VLA‐4, α4β1, CD49d, and CD29) integrin on leukocytes. VLA‐4 is highly expressed on T cells and neutrophils and is absent on blood‐mono. hiPSC‐mono show increased VLA‐4 expression so that use of optimal stimulation conditions that facilitate VCAM‐1 upregulation on hiPSC‐ECs is recommended. Alternatively, HUVECs can be used, as they are known to express high levels of VCAM‐1 after TNF‐α treatment. hiPSC‐ECs used in this assay were differentiated and cultured as previously described (Orlova et al., 2014a; 2014b).

Macrophages show diverse functions in vivo including phagocytosis, microbicidal killing, cytokine production, antigen presentation, and antitumor activity (Woods, Lu, Ceddia, & Lowder, 2000). Functional activities of iPSDM subtypes can be assessed using various cell‐type‐specific assays. Endocytotic activity of iPSDMs can be determined using AcLDL uptake (Fig. 4A and 4B). Phagocytosis of bacteria is studied by adding green fluorescent protein (GFP)‐labeled *Escherichia coli* to iPSDMs and the phagocytic efficiency is quantified by FACS (Fig. 4C and 4D). The capacity for uptake of apoptotic cells (efferocytosis) can also be measured. Target cells induced to undergo apoptosis by UV light exposure are added to iPSDMs and the efferocytosis efficiency quantified by FACS (Fig. 5A). Different target cell types can be used for efferocytosis including hiPSCs and blood neutrophils although cancer cells are best avoided due to high tumor phagocytosis by macrophages. Lastly, tumor phagocytosis can be measured to investigate the anti‐tumor activity of iPSDMs. Jurkat cells are often used as target cells for this; they are incubated with IPSDMs in the presence or absence of CD47 blocking antibody (anti‐CD47) and the phagocytic efficiency is quantified by FACS (Fig. 5B). Other cancer cell types which express high levels of CD47 can also be used in the assay. PBDMs that differentiate and polarize in the same way as iPSDMs can be included as controls for the characterization and functional assays of iPSDMs.

iPSDMs derived from this protocol showed higher endocytosis and efferocytosis capacities than PBDMs (Cao et al., 2019), indicating a more tissue‐resident macrophage‐like identity (A‐Gonzalez et al., 2017; Swirski, Robbins, & Nahrendorf, 2016). In addition, it has been shown that iPSDMs can be conditioned by neuronal cells to acquire a microglia identity in vitro (Haenseler et al., 2017; Takata et al., 2017). Interleukin 34 (IL‐34) could also drive the microglia differentiation from hiPSCs (Brownjohn et al., 2018; Muffat et al., 2016). These results suggest that the iPSDMs we obtain can be a source of tissue resident macrophages, especially for microglia cells. The characterization methods and functional assays described in this protocol could also be applicable for other source of monocytes and macrophages, as they had been tested with both iPSDMs and PBDMs previously (Cao et al., 2019).

### Critical Parameters

hiPSCs used for the differentiation in this protocol are cultured on vitronectin‐coated plates in defined TeSR‐E8 medium. Compared to other methods using irradiated mouse embryonic fibroblast (MEF) feeders or FBS, this defined protocol is simpler and more robust. Spontaneous differentiation of hiPSCs should be avoided as an undifferentiated state of hiPSCs is required for the successful differentiation of monocytes and macrophages. Cells should be passaged regularly every week and successful maintenance of hiPSCs should yield uniform and compact colonies with >90% confluency after 1 week of passaging.

The whole differentiation process consists of two parts: Derivation of monocytes from hiPSCs in 14 to 15 days and the differentiation of mature macrophage subtypes from cryopreserved monocytes in 6 to 7 days (Fig. 1). Compared to the protocol published originally (Cao et al., 2019), we included a flexible time for the induction of HPCs to monocytes, as well as for the differentiation of M0 macrophages from monocytes; this can be adjusted depending on each differentiation and hiPSC line used. The monocyte induction from day 9 should last 5 to 6 days in order to get enough CD14+ monocytes with >50% purity before isolation. However, a longer induction time than 6 days should be avoided as monocytes usually start to adhere and differentiate into macrophages from day 14 or 15.

The initial seeding density of hiPSCs on Matrigel is absolutely crucial for the efficient differentiation of hematopoietic cells from hiPSCs. Too high seeding densities could inhibit or abolish completely the production of roundish HPCs from day 5 to 9. At the beginning, we recommend testing different seeding densities with split ratios ranging from 1:30 to 1:50, in order to find out the optimal seeding density of the hiPSC line used.

The dissociation step on day 9 of the differentiation is one of the most labor intensive and critical steps during the whole differentiation process. Cells on day 9 contain multilayers including roundish HPCs on the top and other stromal or progenitor cells at the bottom. We recommend dissociating and collecting the majority of the cells by dissociation with enzyme and scraping. However, the whole procedure should be gentle to avoid too much cell death, as dead cells could inhibit differentiation and lead to activation of monocytes and reduction of the yield.

Both freshly isolated and cryopreserved monocytes can be used for the induction of functional macrophages on FBS‐coated plates. We strongly recommend using fresh monocytes when a large quantity of macrophages is needed, due to a much higher proliferative and recovery rate of fresh monocytes after seeding compared to cryopreserved monocytes. However, cryopreserved monocytes are excellent for disease modeling and other biological studies where macrophages from multiple batches and hiPSC lines are needed for experiments.

With regards to supplementation with M‐CSF during the differentiation, we used 80 ng/ml for both monocyte induction from day 9 to day 14 or 15 and later, the macrophage differentiation from monocytes. We observed higher M‐CSF concentrations (up to 80 ng/ml) improved the differentiation efficiency of CD14+ monocytes. However, lower concentrations of M‐CSF (40 to 80 ng/ml) can be used for macrophage induction from monocytes without affecting the yield and cell activity.

During the development of our protocols for the polarization of M1 and M2 macrophages, we found a higher starting density of M0 could benefit the survival of polarized M1 and M2 macrophages. So, a higher seeding density of monocytes and longer differentiation time for M0 are recommended when large numbers of polarized M1 and M2 are needed. The optimal polarization time is 24 to 48 hr. M2 macrophages start to undergo apoptosis after 48 hr of polarization. So, we recommend polarizing M1 and M2 macrophages from M0 for not more than 48 hr.

### Troubleshooting

The detailed troubleshooting guidelines can be found in Table 1.

**Table 1:** Troubleshooting

Step	Problem	Possible reason	Solution
Basic Protocol 1 (step 18)	Very few roundish hematopoietic cells	Starting seeding density of hiPSCs is too high	Reduce hiPSC seeding density
Basic Protocol 1 (step 28)	Most monocytes adhere to the plate	Too many dead cells introduced on day 9 by dissociation	During dissociation, pipet cells gently and reduce incubation time with Accutase solution; Harvest monocytes on day 14 instead of day 15
Support Protocol 1 (step 10)	Liquid does not flow through the column	Column gets blocked by cell clumps	Filter cells with CellTrics® filter to get single cell suspension
Support Protocol 2 (step 8a)	Too high or low cell density	Cell suspension is not mixed well before loading; Too much or few cell suspensions loaded	Mix cells well before loading; Optimize volume of cell suspension loaded
Basic Protocol 2 (step 6)	No cells adhere 2 days after thawing	Old IF9S medium; Cell death during thawing procedure	Prepare fresh IF9S; Avoid vigorous pipetting and minimize time of thawing procedure
Support Protocol 4 (step 6c)	Too few apoptotic cells or too many necrotic cells	UV exposure time not optimal; Incubation time after UV treatment is not optimal	Adjust UV exposure time from 3 to 7 min; Adjust incubation time from 1 to 2 hr

### Understanding Results

As the initial step of the differentiation protocol, seeding of hiPSCs at an optimal density is crucial for the efficient induction of hematopoietic cells. Around 60,000 hiPSCs should be seeded in each well of the 6‐well plate which should give rise to 30 to 40 small size colonies the next day. These colonies expand continuously from day 0 to day 5 (Fig. 1). More than 60% of CD140a+ mesoderm cells on day 2 and 40% VEC+ endothelium on day 5 should be obtained and most of these VEC+ endothelium on day 5 should also be CD73‐HEs (Fig. 1). Non‐adherent, roundish hematopoietic cells should appear from the center of colonies from around day 7 and their number should grow continuously until day 9 (Fig. 1 and Supporting Information Video 1). The number of roundish HPCs on day 9 is an easy and reliable way to assess the hematopoietic differentiation efficiency. More than 50% of the cells on day 9 should be CD43+ HPCs. Differentiations with few or no HPCs on day 9 are regarded as failed and should not be continued (see Table 1 for troubleshooting). On day 9, all floating cells and adherent cells are collected and seeded in the low‐attachment plate. Large numbers of single roundish cells and dark spheres can be observed under the microscope. During suspension culture from day 9 to day 14 or 15, the total cell number and cell morphology will hardly change. In some differentiations, adherent cells can already be observed on day 14, indicating activation and maturation of monocytes that are ready to be harvested. For most differentiations, cells should be ready for harvest on day 15 when >50% have become CD14+ monocytes. More than 10 million CD14+ monocytes should be obtained from each 24‐well low‐attachment plate and the purity of CD14+ cells should be >90% after isolation (Fig. 1; Cao et al., 2019).

An advantage of this protocol is that hiPSC‐mono can be cryopreserved yet retain their phenotype and functionality. The recovery rate of cryopreserved hiPSC‐mono should be >40%. Thawed monocytes express similar levels of CD14, CD45, CD49d, CD18, CD29, and CD11b as newly isolated hiPSC‐derived monocytes (Fig. 2A). To assess their functionality, a microfluidic assay can be performed with thawed hiPSC‐mono (Fig. 2B). Thawed hiPSC‐mono should adhere to both hiPSC‐ECs and HUVECs activated by TNF‐α, while greater adhesion should be observed with HUVECs than hiPSC‐EC (Fig. 2C and 2D).

Thawed hiPSC‐mono can also be polarized and give rise to M0, M1, and M2 macrophages. After 48 hr of seeding on FBS‐coated plates, only a minority of thawed monocytes will adhere with a roundish morphology. After refreshing on day 2, cells proliferate rapidly and reach ∼80% confluence on day 4 or day 5 depending on the performance of differentiation and cryopreservation. After polarization for another 2 days, M0 should show an elongated morphology while M1 have a stellar shape with multiple protrusions and M2 have a more rounded morphology with a lower cell density compared to M0 and M1 (Fig. 1). All subtypes should express comparable levels of specific pan‐macrophage markers CD11b, CD18, and CD45 by FACS. M0 and M2 express higher CD206 and CD163, while M1 have the highest CD80 expression (Fig. 3). Optionally, reverse transcription‐quantitative polymerase chain reaction (RT‐qPCR) and multiplex cytokine assay can be performed for further characterization of derivative macrophage subtypes (Cao et al., 2019).

To investigate the functional activities of iPSDMs, several assays described in this protocol can be applied based on individual research interests. In the endocytosis assay, all iPSDM subtypes should show high levels of Dil‐AcLDL uptake and higher endocytosis activity should be observed in M0 and M2 than M1 (Fig. 4A and 4B). In the bacterial phagocytosis assay, anti‐inflammatory M0 and M2 should show higher phagocytic activity of *E. coli* than the pro‐inflammatory M1 subtype (Fig. 4C and 4D). For the efferocytosis assay, iPSDMs should take up more apoptotic cells than live cells that have not undergone UV treatment (Fig. 5A). Regarding the apoptotic target cells used in the efferocytosis assay, >50% Annexin V+PI‐ apoptotic cells should be induced by the UV treatment (Cao et al., 2019). To investigate tumor phagocytotic activity of iPSDMs, CFSE‐labeled Jurkat cells can be added to iPSDMs. After incubation, CFSE‐labeled Jurkat cells should be observed within iPSDMs cell bodies under the microscope. iPSDMs should show significantly higher phagocytosis of Jurkat cells in the presence of anti‐CD47 compared to the control group without anti‐CD47 blocking antibody (Fig. 5B).

### Time Considerations

#### Basic Protocol 1

The differentiation of monocytes from hiPSCs takes 14 to 15 days in total.

Steps 1 to 11: ∼20 min will be need for passaging of hiPSCs for maintenance.

Steps 12 to 15: ∼20 min will be needed for passaging of hiPSCs to Matrigel‐coated plate for differentiation.

Steps 16 to 18: ∼15 min will be needed on each day of day 0, 2, 5, 7 to change the media.

Steps 19 to 26: ∼1 hr will be needed to collect floating hematopoietic cells and dissociation of adherent cells.

Step 27: ∼15 min will be needed to change medium for the suspension culture.

#### Support Protocol 1

Steps 1 to 15: ∼1.5 hr will be needed to collect all cells, perform CD14+ cell isolation, and cryopreservation of isolated cells.

#### Support Protocol 2

Steps 1a to 8a: ∼3 hr will be needed to do Wright‐Giemsa staining (include 2 hr for drying of slide).

Steps 1b to 6b: ∼1 hr will be needed for FACS of monocytes (include 30 min incubation).

#### Basic Protocol 2

The differentiation of macrophage subtypes from monocytes takes 6 to 7 days in total.

Steps 1 to 5: ∼15 min will be needed to thaw cryopreserved monocytes and seed into FBS‐coated plate.

Step 6: ∼10 min will be needed to change the medium 2 days after seeding.

Steps 8 to 10: ∼20 min will be needed for the dissociation and seeding of M0 macrophages.

Step 11: ∼10 min will be needed to change the medium.

#### Support Protocol 3

Steps 1a to 6a: ∼1.5 hr will be needed for FACS of iPSDMs (include 30 min incubation).

Steps 1b to 12b: ∼6 hr will be needed for multiplex cytokine assay (include 3 hr incubation).

#### Support Protocol 4

Steps 1a to 6a: ∼5.5 hr will be needed to perform the AcLDL uptake assay (include 4 hr incubation).

Steps 1b to 9b: ∼2.5 hr will be needed to perform the bacterial phagocytosis assay.

Steps 1c to 14c: ∼4 hr will be needed to perform the efferocytosis assay (include two times, 1 hr incubation).

Steps 1d to 11d: ∼5 hr will be needed to perform the tumor phagocytosis assay (include 2 hr incubation).

## Supporting information

Video S1: Time‐lapse video from differentiation day 7 to day 9 Video legend: Time lapse take from differentiation day 7 to day 9 at a frequency of 30 min each frame. This video plays at 10 frames per seconds. Scale bar represents 200 μm.
